# Antimicrobial susceptibility pattern of pathogens isolated from surgical wound infections in tertiary care hospitals of Pakistan

**DOI:** 10.4314/ahs.v25i1.2

**Published:** 2025-03

**Authors:** Rashid Iqbal, Palwasha Ahmad, Zumaira Tahir

**Affiliations:** 1 Department of Health Sciences Technology, National Skills University, Islamabad, Pakistan; 2 Department of Social Sciences and Humanities, Bahria University Islamabad, Pakistan; 3 Shifa College of Medical Technology, Shifa Tameer-e-Milat University, Islamabad, Pakistan

**Keywords:** Antibiotics, gram-positive, gram-negative, sensitive, resistant

## Abstract

**Background:**

Antimicrobial susceptibility testing for post-surgical site infections is crucial amid rising cases and antibiotic resistance.

**Objective:**

This study aimed to evaluate demographic factors, the occurrence of surgical wound site infections, pathogens associated with these infections, and antimicrobial susceptibility of the isolated pathogens.

**Subjects and Methods:**

A cross-sectional study including 384 patients suffering from post-surgical site infections was conducted in tertiary care hospitals in Pakistan over six months.

**Results:**

The study participants' ages ranged from 7 to 74 years old, with a mean age (±SD) of 30.4 (±9.5) years, and 44.8% of them were female. Among 384 study subjects, bacterial pathogens were isolated from 295 (76.80%). The frequency of gram-negative was 58.75%, whereas the frequency of gram-negative was 41.25%. Methicillin-resistant Staphylococcus aureus (MRSA), (19%), Staphylococcus aureus (Staph aureus) (18.0%), and Escherichia coli (E. coli) (18.0%) were the most common organisms isolated from wound infections. A significant association was present between occupation and culture sensitivity with a p-value of 0.01. Most of the culture-positive population had appendectomy site infection (92.90%). MRSA had the highest sensitivity to vancomycin (89.8%) and the highest resistance to gentamicin (85.7%). Staph. aureus was most sensitive to imipenem (80%) and most resistant to gentamicin (68.4%). E. coli was most sensitive to imipenem (100%) and most resistant to ceftazidime (90%).

**Conclusion:**

This study has provided a thorough description of the prevalence of gram-positive and gram-negative bacteria, as well as their relationships to wound type and demographic parameters.

## Introduction

Infections from surgical wounds are the main source of hospital-acquired infections worldwide, they account for 38% of hospital-acquired infections in the United States, there are almost 2-5% of the patients whose surgeries get affected. Post-surgical infections (PSSIs) are infections induced by a surgical intervention at the site of incision within thirty days after the surgery or within ninety days if prosthetic implantation is done during the procedure as stated by the Centers for Disease Control and Prevention^1^. Despite developments in infection control practices such as improved theater ventilation, sterilization procedures, and effective antimicrobial prophylactic use, PSSIs continue to be a primary cause of morbidity and mortality^2^. Predisposing factors for the development of post-surgical infections include the extent of microbiological contamination, patients' age, the time taken during the operation, and the history of diabetes, obesity, or immunosuppression, various research indicates that Staph. aureus and gram-negative bacilli such as Escherichia coli, Klebsiella & Pseudomonas play major roles as a source of post-surgical infections^3^. The occurrence of post-surgical infections due to the bacteria that have developed antimicrobial-resistant species, for instance, Methicillin Resistant Staph. aureus (MRSA) & Vancomycin Resistant Staph. aureus (VRSA) is on the rise. This has also increased the overall medical cost because of the prolonged hospital stay of patients with infected wounds^4^. Antibiotiprophylaxis is recommended for surgical operations involving artificial devices and prostheses, as well as for contaminated and uncontaminated surgeries. Due to weakened immunity or heightened vulnerability before and after significant surgeries, such as neurosurgery, open-heart surgery, or ocular surgery germs can develop resistance to certain medications., strict hygiene measures become even more crucial in these situations to minimize the risk of infections or complications. Antimicrobial susceptibility testing plays a vital role in determining bacteria's susceptibility to antibiotics, enabling clinicians to initiate appropriate medications^5^. This research aims to establish the volume, distribution, and antibiotic susceptibility profiles of bacterial pathogens isolated from post-operative wounds in public hospitals in Pakistan.

## Subjects and Methods

### Study setting

The study was conducted at the Microbiology Department of Holy Family Hospital (HFH), Rawalpindi, Pakistan Institute of Medical Sciences (PIMS), Islamabad, Pakistan. The duration of the study was 6 months from August 2022 to February 2023. The institute's Institutional Review Committee granted ethical approval. (IRB# 202-22).

### Study Design and Population

A cross-sectional survey was carried out. Inclusion criteria comprised 384 individuals who were hospitalized during the study period and had surgery as either elective or emergency surgical operations. Exclusion criteria comprised of the patients who underwent another procedure within a month, contracted an infection before the research period, and patients who were receiving antibiotics after the procedure. The sample size was determined using the Open Epi calculator^6^. The convenience sampling method was employed. A structured, & content-validated questionnaire has been utilized to collect the data.

### Wound Swab Sample Collection

According to Levine's technique, patients with open wounds were examined and affected area was prepared for collecting the sample, During the site preparation the surface was cleaned with moist sterile gauze and sterile normal saline solution (NS)^7^. Swabs from the affected site were taken and aseptic techniques were used. cotton swab spun vigorously enough after bathing the location with an antiseptic solution. Wound specimens were transferred within thirty minutes from the collecting site to the microbiology lab by putting swabs in sterile test tubes containing half mL of sterile NS^8^.

### Culture and identification of isolates

Each sample underwent an inoculation concurrently on plates of mannitol salt agar, blood agar, and MacConkey agar plates (Oxoid Ltd), and was kept in incubation for 24 hours in an aerobic environment at 37 °C. If mixed colonies were created on agar plates, they were subcultured onto blood and MacConkey agar plates and kept for incubation twenty-four to forty-eight hours at 37° Celsius. Isolated bacteria were identified using common microbiological procedures such as colonial morphology, Gram's staining reaction, and biochemical test series^9^.

Pure colonies from cultures were subjected to colony morphology, gram stain^10^ and biochemical testing to determine the isolates' ultimate identification^11^.

### Antibiotic Susceptibility Testing

Pure colonies from each isolated culture were suspended in sterile N/S solution and kept in incubators at 37 °C for at least fifteen minutes. The suspension was scaled down to 0.5 MacFarland standard. The suspension was applied to Mueller-Hinton agar (Oxoid Ltd) utilizing a disinfected cotton tip application stick. The improved Kirby-Bauer disc diffusion technique was then used to figure out the susceptibility profile of antibiotics^12^. After identifying the isolates of bacteria, a clinical and laboratory standard institute (CLSI) recommended Drug susceptibility test (DST) conventional disc diffusion technique was carried out. For Gram positive isolates, the following medications were used: cefazolin (30 µg), erythromycin (15 µg), clarithromycin (15 µg), amoxicillin/clavulinate (30 µg), Amoxicillin (30 µg), imipenem (10µg), meropenem (10 µg), Gentamicin (10 µg), cefoperazone (75 µg), penicillin (10 µg) and vancomycin (30 µg). Gram negative isolates were tested with amikacin (30 µg), aztreonam (30 µg), ciprofloxacin (5 µg), ceftazidime (30 µg), cotrimoxazole (25 µg), carbenicillin (100 µg), Gentamicin (10 µg), Ampicillin (10 µg), pipracillin (30 µg), imipenem (10µg) and meropenem (10 µg)^12^.

### Methicillin-resistant Staphylococcus

Aureus was identified using cefoxitin (MRSA) (30 mcg). MRSA was defined as Staphylococcus aureus with a region of inhibition measuring 21 mm with cefoxitin on Mueller Hinton Agar following a period of overnight incubation at 37 °C^13^.

### Quality Control

The produced media's efficiency was evaluated by introducing quality control bacteria, Staph. aureus (ATCC-25923) and E. coli (ATCC-25922). Furthermore, sterility was tested by incubation of 5% of the prepared medium at 37 °C for 24 to 48 hours, and gram staining and biochemical testing reagents were checked by standardized strains of Staphylococcus aureus and Escherichia coli^13^.

## Results

The study participants' ages ranged from 7 to 74 years old, with a mean age (±SD) of 30.4 (±9.5) years, and 44.8% of them were female. According to Table 1, around 59.7%, 20.1%, 15.6%, 26%, and 24% of the participants lived in metropolitan regions, were merchants, students, illiterate, and had a high school education respectively. A total of 295 (76.8%) of the 384 samples taken from wound infections showed aerobic organism growth, whereas 89 (23.2%) exhibited no growth. The overall count of bacterial isolates was 303, as eight (2.6%) specimens indicated the existence of two distinct bacteria.

**Table 1:** The association between sociodemographic characteristics of study participants and culture positivity from wound samples

Characteristic, n (%)	Culture positive	Culture Negative	Total	p-value
Age				0.31
1-19	64 (74.6)	23 (26.4)	87 (22.7)	
20-35	24 (75)	8 (25)	32 (8.3)	
36-50	54 (70.1)	23 (29.9)	77 (20.1)	
51-60	62 (82.6)	13 (17.4)	75 (19.5)	
>61	91 (80.5)	22 (19.5)	113 (29.4)	
Total	295 (76.9)	89 (23.1)	384 (100)	

Gender				0.23
Male	158 (74.5)	54 (25.5)	212 (55.2)	
Female	137 (79.7)	35 (20.3)	172 (44.8)	
Total	295 (76.9)	89 (23.1)	384 (100)	

Residence				0.47
Urban	173 (75.5)	56 (24.5)	229 (59.7)	
Rural	122 (78.7)	33 (21.3)	155 (40.3)	
Total	295 (76.9)	89 (23.1)	384 (100)	

Occupation				0.01
Self-employee				
Government	38 (88.4)	5(11.6)	43 (11.2)	
employee	34 (87.2)	5 (12.8)	39 (10.2)	
Day-laborer	29 (74.4)	10 (25.6)	39 (10.2)	
Merchant	59 (76.6)	18 (23.4)	77 (20.1)	
Farmer	36 (90)	4 (10)	40 (10.4)	
Housewife	22 (71)	9 (29)	31 (8.0)	
Student	39 (65)	21 (35)	60 (15.6)	
Unemployed	38 (69)	17 (31)	55 (14.3)	
Total	295 (76.9)	89 (23.1)	384 (100)	

Education status				0.74
Illiterate	75 (75)	25 (25)	100 (26)	
Preschool	45 (71.4)	18 (28.6)	63 (16.4)	
Elementary	59 (78.7)	16 (21.3)	75 (19.5)	
High school	73 (79.3)	19 (20.7)	92 (24)	
College and above	43 (80)	11 (20)	54 (14.1)	
Total	295 (76.9)	89 (23.1)	384 (100)	

Out of 303 isolates, 125 (41.25%) were gram-positive while 178 (58.75%) were gram-negative as shown in figure 01.

The study included participants with diverse surgical backgrounds, comprising 15.7% from perianal fistula operation, 15.1% from incisional hernia repair, 6.8% from umbilical and incisional hernia repair, and 6.5 % from abdominal laparotomy as shown in the table 02.

**Table 2:** Prevalcence of infections in post surgical wound site infections

Characteristics, n (%)	Culture positive	Culture Negative	Total
Wound type			
Perianal fistula operation	39 (67.2)	19 (32.8)	58 (15.1)
Incisional hernia repair	49 (82.7)	11 (18.3)	60 (15.7)
A bone knee amputation	12 (80)	3 (20)	15 (3.9)
Laparoscopic cholecystectomy	14 (87.5)	2 (12.5)	16 (4.2)
Abdominal laparotomy*	21 (84)	4 (16)	25 (6.5)
Right leg skin graft	6 (75)	2 (25)	8 (2.1)
Appendectomy	13 (92.9)	1 (7.1)	14 (3.6)
Umbilical and incisional hernia repair	18 (69.2)	8 (30.8)	26 (6.8)
Groin abscess incision and drainage	8 (72.8)	3 (27.2)	11 (2.9)
Perianal surgery	8 (66.7)	4 (33.3)	12 (3.1)
Right gluteal abscess	14 (70)	6 (30)	20 (5.2)
Perianal fistulectomy	11 (78.6)	3 (21.4)	14 (3.6)
Perianal abscess	5 (62.5)	3 (37.5)	8 (2.1)
Back lipoma excision	5 (100)	0 (0)	5 (1.3)
Right inguinal hernia repair	11 (68.8)	5 (31.2)	16 (4.2)
Hand surgery	6 (85.7)	1 (14.3)	7 (1.8)
Below knee amputation	11 (100)	0 (0)	11 (2.9)
Debridement of heel ulcer	13 (61.9)	8 (38.1)	21 (5.5)
Debridement and skin graft	9 (100)	0 (0)	9 (2.3)
Vertebral fixation	4 (100)	0 (0)	4 (1.0)
Right-forearm graft	5 (100)	0 (0)	5 (1.3)
Right-thigh operation	13 (68.4)	6 (31.6)	19 (4.9)
Total	295 (76.9)	89 (23.1)	384 (100)

* Abdominal laparotomy due to intestinal obstruction

### Abdominal laparotomy due to intestinal obstruction

The results of antimicrobial susceptibility patterns showed that a variety of antibiotics may be used to treat Gram-positive pathogens. Vancomycin (89.8%) was the most effective treatment for MRSA. Enterococcus showed susceptibility to imipenem (100%) (Table 03).

**Table 3:** Antibiotic efficacy in infections caused by Gram-positive bacteria

Antibiotics	Susceptibility	MRSA	Enterococcus	Staph. aureus	Total	P-value
Cefazolin	Sensitive	9 (56.3)	2 (66.7)	7 (77.8)	18 (64.3)	0.45
	Resistant	7 (43.7)	1 (33.3)	2 (22.2)	10 (35.7)	
	Total	16 (100)	3(100)	9(100)	28 (100)	

Erythromycin	Sensitive	3 (50)	4 (66.7)	9 (60)	16 (59.3)	0.46
	Resistant	3 (50)	2 (33.3)	6 (40)	11 (40.7)	
	Total	6(100)	6 (100	15 (100)	27 (100)	

Clarithromycin	Sensitive	3 (30)	3 (42.9)	15 (75)	21 (56.8)	0.07
	Resistant	7 (70)	4 (57.1)	5 (25)	16 (43.2)	
	Total	10 (100)	7 (100)	20 (100)	37 (100)	
Imipenem	Sensitive	5 (55.6)	2 (100)	8 (80)	15 (71.4)	0.32
	Resistant	4 (44.4)	0 (0)	2 (20)	6 (28.6)	
	Total	9(100)	2(100)	10 (100)	21 (100)	

Meropenem					19 (63.3)	0.77
	Sensitive	9 (64.3)	2 (66.7)	8 (61.5)	11 (36.7)	
	Resistant	5 (35.7)	1 (33.3)	5 (38.5)	30	
	Total	14 (100)	3(100)	13 (100)	(100)	

Gentamicin	Sensitive	2 (14.3)	0 (0)	12 (31.6)	14 (24.6)	0.17
	Resistant	12 (85.7)	5 (100)	26 (68.4)	43 (75.4)	
	Total	14 (100)	5(100)	38 (100)	57 (100)	

Cefoperazone	Sensitive	33 (62.3)	5 (62.5)	30 (56.6)	68 (59.6)	0.82
	Resistant	20 (37.7)	3 (37.5)	23 (43.4)	46 (40.4)	
	Total	53 (100)	8(100)	53 (100)	114 (100)	

Vancomycin	Sensitive	44 (89.8)	2 (66.7)	20 (80)	66 (85.7)	0.32
	Resistant	5 (10.2)	1 (33.3)	5 (20)	11 (14.3)	
	Total	49 (100)	3(100)	25 (100)	77 (100)	

When assessing the antimicrobial susceptibility of gram-negative pathogens, a range of antibiotics also demonstrated effectiveness in potential treatment options for these infections. The most sensitive antibiotics against E.coli, Pseudomonas aeruginosa, Pseudomonas species, Acinetobacter species, Klebsiella species, Proteus species and Coliform were imipenem (100%), imipenem (77.8%), amikacin (77.8%), amikacin (94.7%), meropenem (80%), pipracillin (75%), and ampicillin (75%) respectively (Table 04).

**Table 4:** Antibiotic efficacy in infections caused by gram-negative bacteria

Antibiotics	Susceptibility	E.coli	Pseudomonas aeruginosa	Pseudomonas species	Acinetobacter species	Klebsiella species	Proteus species	Coliform	Total	p-value
Amikacin									109	0.00
		44					9 (64.3)		(71.2)	
	Sensitive	(91.6)	18 (66.7)	14 (77.8)	18 (94.7)	7 (63.6)	5 (35.7)	9 (56.3)	44 (28.8)	
	Resistant	4 (8.4)	9 (33.3)	4 (22.2)	1 (5.3)	4 (36.4)	14	7 (43.7)	153	
	Total	48 (100)	27 (100)	18 (100)	19 (100)	11 (100)	(100)	16 (100)	(100)	

Aztreonam	Sensitive	1 (33.3)	7 (33.3)	1 (7.1)	0 (0)	2 (66.7)	0 (0)	2 (50)	9 (22)	0.34
	Resistant	2 (66.7)	14 (66.7)	13 (92.9)	2 (100)	1 (3.33)	1 (100)	2 (50)	32 (78)	
	Total	3 (100)	21 (100)	14 (100)	2 (100)	3 (100)	1 (100)	4 (100)	41 (100)	

Ciprofloxacin		26 (49.1)					10 (66.7)		90 (52.9)	0.78
	Sensitive	27	16 (57.1)	11 (50)	13 (54.2)	7 (63.6)	5 (33.3)	7 (41.2)	80 (47.1)	
	Resistant	(50.9)	12 (42.9)	11 (50)	11 (45.8)	4 (36.4)	15	10 (58.8)	170	
	Total	53 (100)	28 (100)	22 (100)	24 (100)	11 (100)	(100)	17 (100)	(100)	

Ceftazidime									32 (19.4)	
							3 (20)		133	
	Sensitive	5 (10)	10 (38.5)	3 (14.3)	4 (22.2)	3 (20)	12 (80)	4 (20)	(80.6)	
	Resistant	45 (90)	16 (61.5)	18 (85.7)	14 (77.8)	12 (80)	15	16 (80)	165	
	Total	50 (100)	26 (100)	21 (100)	18 (100)	15 (100)	(100)	20 (100)	(100)	0.15

Gentamicin		18 (33.3)					6 (42.9)		51 (30.9)	
	Sensitive	36	8 (27.6)	5 (29.4)	4 (19)	4 (33.3)	8 (57.1)	6 33.3)	114 (60.1)	
	Resistant	(66.7)	21 (72.4)	12 (70.6)	17 (81)	7 (66.7)	14	12 (66.7)	165	
	Total	54 (100)	29 (100)	17 (100)	21 (100)	12 (100)	(100)	18 (100)	(100)	0.84

Imipenem	Sensitive	15 (100)	7 (77.8)	3 (75)	6 (66.7)	4 (80)	3 (75)	5 (62.5)	43 (79.6)	
	Resistant	0 (0)	2 (22.2)	1 (25)	3 (33.3)	1 (20)	1 (25)	3 (37.5)	11 (20.4)	
	Total	15 (100)	9 (100)	4 (100)	9 (100)	5 (100)	4 (100)	8 (100)	54 (100)	0.38

Meropenem	Sensitive	8 (47.1)	6 (66.7)	2 (50)	4 (50)	4 (80)	2 (66.7)	5 (71.4)	31 (58.5)	
	Resistant	9 (52.9)	3 (33.3)	2 (50)	4 (50)	1 (20)	1 (33.3)	2 (28.6)	22 (41.5)	
	Total	17 (100)	9 (100)	4 (100)	8 (100)	5 (100)	3 (100)	7 (100)	53 (100)	0.80

## Discussion

Surgical wound site infection is one of the most dreaded complications during the post-operative management of wounds. In the absence of adequate care, the surgical site wound is a perfect medium for the colonization and reproduction of all types of pathogens.

The skin that usually protects against microbes by acting as a physical barrier, is disrupted during surgery, hence the surgical site becomes the entry point for microbial invasion^14^.

In the present study, MRSA (19%), S. aureus (18.0%), and E. coli (18.0%) were the most common organisms isolated from wound infections. Several studies carried out formerly on wound infection from various parts of the globe showed that S. aureus and E. coli were the predominant isolates^15^. The high frequency of S. aureus infection could be due to its endogenous nature. With the disruption, the skin also leads to infection from S. aureus as is common on skin surfaces^16^.

A significant association was present between occupation and culture sensitivity with a p-value of 0.01. From culture, the population majority is from the category of farmers (90%), self-employed (88.4%) and Government employees (87.2%). The association between culture sensitivity and the rest of the demographic characteristics was insignificant statistically. Higher culture positivity in farmers was also reported by another study in literature^17^.

The association between wound type and culture sensitivity was insignificant. Most of the culture-positive population had appendectomy site infection (92.90%) which was followed by laparoscopic cholecystectomy site infection (87.50%). A meta-analysis that included studies from 49 countries also reported a high burden of surgical site infection after appendectomy, especially in Africa and low income-countries^18^.

Across all gram-positive bacteria most sensitive to vancomycin (85.70%) while they were most resistant to gentamicin (75.40%). Almost, a similar pattern of sensitivity was also observed in a study in literature. Significant susceptibility of gram-positive bacteria to vancomycin in the study population could be due to lesser use of these antibiotics as these antibiotics are costly^19^.

Overall, gram-negative bacteria were most sensitive to imipenem (79.6%) followed by amikacin (71.2%) while they were most resistant to ceftazidime (80.6%) followed by ceftriaxone (80.3%). Other studies have also noted the remarkable susceptibility of gram-negative bacteria to imipenem and amikacin^20^.

This study has greatly described the prevalence of both gram-positive and gram-negative bacteria along with their association with demographic factors and wound type. Most importantly the susceptibility of various bacteria to different antibiotics.

## Figures and Tables

**Figure 1:**
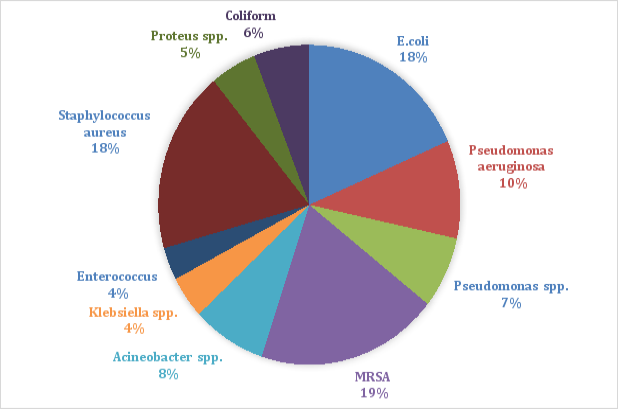
Distribution of isolated gram-positive and gram-negative bacteria from surgical wound infections
